# The Formation of Blood-Corpuscles

**Published:** 1882-05

**Authors:** 


### The Formation of Blood-Corpuscles.

The authority with which M. Malassez speaks upon all subjects connected with the constitution of the blood confers importance on a summary of facts relating to the formation of red blood-corpuscles in bone marrow, which he has recently communicated to the Societe de Biologie. This summary contains an account of the results attained in a series of investigations on the subject, and embody facts and theories of undeniable importance. It is generally admitted, since the discoveries of Neumann and Bizzozero, that the red cells discovered by the former in the bone marrow of the mammalia are embryonal corpuscles, but various opinions are held regarding the origin of these cells and the mode in which they are transformed into red blood discs. It is to these two points that his investigations have been especially directed. The first hypothesis which was advanced—and it is still accepted by many authorities—is that the red cells are transformed into corpuscles by the gradual disappearance of their nuclei. If the process is studied in animals in which the red corpuscles are nearly the same size as these cells, forms of the latter may be observed in which the nuclei appear to be in process of disappearance. But if methods are employed which leave the structure of the cells unaffected, and especially if the examination is made in animals in which the red blood discs are considerably smaller than these cells, such intermediate forms cannot, according to Malassez, be discovered. Moreover, the corpuscle is so much smaller than the cell that a transformation of one into the other would imply either a contraction of the protoplasm of the cell, or a change by which this breaks up and forms more than one globule, and each assumption is destitute of evidence. Hence the conclusion is drawn that this theory of transformation by destruction of the nucleus is untenable. Rindfleisch, from appearances which he observed in some cells, concluded that the change does not consist in a destruction of the nucleus, but in the exit of this from the cell. This appearance is stated by Obrastzow to be due to an alteration of the cells analogous to that which Donne long ago noted in the corpuscles of the frog treated by water. It is probably an artificial phenomenon, for it cannot be observed in recent or well-preserved specimens.

In studying the formation of blood-corpuscles in the spleen, Malassez and Picard observed cells, charged with hæmoglobin, which presented protoplasmic buds having the aspect of red corpuscles, but spherical in shape, and it was suggested that these being detached might become separate blood discs. Malassez has succeeded in finding a similar appearance in preparations of bone marrow, which were made in such a manner as to preserve undisturbed the form and structure of the tissue elements. A fragment of fresh marrow was teased out on the object-bearer without the addition of any regent. Sometimes the glass slide was merely touched with a fragment of marrow. The preparation was then exposed to the vapor of osmic acid. The tissue elements being thus fixed, they were colored with picro-carmine, or with eosin and log-wood. In the hare the hæmoglobic cells are very large in proportion to the red corpuscles, and they may present several minute buds. In the rabbit, calf, cat and child the cells are smaller, and usually the bud is single. The most developed prominences tend to become constricted and pedunculated, and thus to assume the appearance of spherical corpuscles. Their substance possesses the same homogeneity, the same refractive power, the same color, and presents the same histo-chemical reactions as do the red blood-corpuscles. The only difference is in their shape. But the normal globules, which are biconcave, may, under certain conditions of humidity, etc., swell up, become concavo-convex, and finally, biconvex and spherical. It does not seem inconceivable, therefore, that these buds, becoming detached, may undergo the converse transformation; and the difference in shape does not constitute adequate ground for the rejection of a theory which can plead in its favor a high degree of intrinsic probability.

Regarding the origin of the red cells of the marrow, certain facts appear to be well established. They may be seen in every stage of nuclear division, and it is therefore generally admitted that they are capable of fissiparous multiplication. But their primary origin is still involved in considerable obscurity. The earliest theory, which is still extensively held, is that they are derived from white blood corpuscles. But the blood contains several varieties of leucocytes. Mallassez believes that we must reject the idea that they come from ordinary leucocytes, with a finely granular protoplasm, and which appear polynucleated when they are treated with osmic acid; and also the view that they are formed from the white corpuscles which stain with eosin, because these elements are completely different from the red cells, and in properly prepared specimens no intermediate forms can be discovered. The origin of these cells from the hyaline leucocytes is much more probable, but is destitute of evidence. Preparations made in the above-described method show, however, a perfect series of cell-forms, at one extremity of which is the red cell, and at the other is an element of very different aspect. The forms which resemble the red cell are clearly more differentiated and specialized than the others, and they may therefore be regarded as more advanced in development. All the forms may be referred to three principal types. Those which are nearest to the hæmoglobic cell differ in their more hyaline protoplasm and smaller amount of hæmoglobin in the reticulated form of their nuclei, and in its slighter affinity for coloring reagents. Another form, further removed from the hæmoglobic cell, possesses a less abundant protoplasm, still less colored and finely granular, a nucleus relatively large, more granular, and tinting still less than those of the last group. In the third form the nucleus occupies the whole, or almost the whole of the cell, as if its substance were diffused through the protoplasm. From the serial character of these forms, Malassez concludes that they, and not the leucocytes of the blood, are the origin of the medullary hæmoglobic cells. The entire succession of forms may be regarded as a progressive development, resulting in the formation of an element which possesses in high degree a special function, that of respiration.

In the animals which possess nucleated red corpuscles the process of formation of these appears to resemble perfectly that of the hæmoglobic cells in animals which possess only non-nucleated blood discs. The cells of origin are the same (and were partially recognized by Bizzozero and Torre), and they present the same transformations, nuclear and protoplasmic. The process does not, however, advance beyond the formation of the red cell. This does not bud, does not form globules; it becomes flattened and passes into the circulation, constituting itself the red corpuscle. Thus the nucleated and non-nucleated red corpuscles are not elements of identical nature, although belonging to the same family and fulfilling the same purpose.

The assertion of Malassez will no doubt, meet with criticism; but he is to be congratulated on having furnished an explanation of the process of blood formation far clearer than any which has yet appeared.—*London Lancet.*

